# Burnout and Health Related Quality of Life among emergency physicians working at emergency at Tertiary Care Hospital in Lahore Pakistan

**DOI:** 10.12669/pjms.39.5.7560

**Published:** 2023

**Authors:** Shahid Sarwar, Asma Mahmood, Mahnoor Farooq Raja, Yasir Mahmud

**Affiliations:** 1 Shahid Sarwar, MBBS, FCPS (Medicine), FCPS (Gastroenterology) MCPS-HPE, FRCP (Edin), Professor of Medicine, Allama Iqbal Medical College Lahore/ Jinnah Hospital, Lahore, Pakistan; 2 Asma Mahmood, MBBS House Officer, Department of Medicine, Allama Iqbal Medical College Lahore/ Jinnah Hospital, Lahore, Pakistan; 3 Mahnoor Farooq Raja, MBBS House Officer, Department of Medicine, Allama Iqbal Medical College Lahore/ Jinnah Hospital, Lahore, Pakistan; 4 Yasir Mahmud, MBBS, FCPS (Medicine) Consultant Physician, Department of Medicine, Allama Iqbal Medical College Lahore/ Jinnah Hospital, Lahore, Pakistan

**Keywords:** Burnout, Emergency, Health related quality of life, Physicians

## Abstract

**Objective:**

To determine frequency of burnout in emergency physicians and to identify its impact on their health-related quality of life (HRQOL).

**Methods:**

In this cross-sectional study, physicians from departments with emergency cover of Jinnah Hospital Lahore were included. Their burnout and HRQOL scores using Maslach Burnout Inventory (MBI) and Short Form (SF)-36 respectively were determined in March 2022. Burnout scores were graded as low, moderate and high and were correlated with domains of HRQOL using chi X^2^ and analysis of variance (ANOVA).

**Results:**

One hundred fifty physicians were included with mean age 26.2 (±2.59), male to female ratio 0.78:1 (66/84) and House Officer (HO) to Postgraduate Resident (PGR) ratio 1.94:1 (99/51). High burnout was identified in 76 (50.7%) participants while 48 (32%) had moderate and 26 (17.3%) had low burnout. Males scored better than female physicians in vitality (0.008), general mental health (0.004), and mental component summary (0.01) domains of HRQOL. Doctors with high burnout had significantly lower scores in both physical component summary (p-value 0.004) and mental component summary (p-value < 0.0001) domains of HRQOL.

**Conclusion:**

Physicians working in emergency settings have high frequency of burnout and it adversely affects their mental and physical health related quality of life.

## INTRODUCTION

Burn out is a psychological condition characterized by emotional exhaustion, depersonalization and declining sense of accomplishment.^1^ Emotional exhaustion is a feeling of being over extended with depletion of emotional and physical resources. Depersonalization is a state of negative, cynical and hostile feelings resulting in emotional detachment in day to day interaction while negative self-appraisal, feeling of incompetence and inefficiency in daily work results in low sense of accomplishment.^2^ Healthcare professionals are twice likely to suffer burnout as compared to other professions with reported incidence of more than 54% in a study of 6880 US physicians.^3^ It results from inherent traits of compulsiveness and self-denial among physicians working in an environment of perfectionism, denial of personal vulnerability and delayed gratification and is precipitated by growing external pressures.^4^

Multiple factors contribute to physician’s burnout which can be work related like excessive workload, long working hours, frequent call duties, need for comprehensive documentations, risk of malpractice suits, added administrative responsibilities and loss of autonomy at work.^5^ Personal factors like sleep deprivation, over commitment and idealism further aggravate sense of burnout among doctors.^6^ Burnout is also worsened by organizational factors like negative leadership behavior, insufficient reward, limited interpersonal collaboration and limited opportunities for progress.^7^ Burnout among physicians results in low job satisfaction, reduced work productivity, low morale, absenteeism, premature retirement and rising incidence of smoking and addiction.^8^ Burnout is much more likely among emergency physicians where it has been reported to be as high as 90% in a Canadian study.^9^

In a study of US physicians, it was noted that among those with excessive work related fatigue, 6.5% have suicide ideations, 10.5% committed medical errors in preceding three months and 3.9% had poor patient safety grades.^10^ Poor health related quality of life (HRQOL) in a burned out physician can explain impaired patient care, increasing medical errors and declining patient satisfaction.^11^

Emergency physicians are mostly post graduate trainees who are more vulnerable to depression and anxiety as well.^12^ However very few studies have tried to determine impact of burnout on HRQOL among emergency physicians who are mostly young fresh graduates working as house officers and post graduate residents and results of these studies are equivocal. We planned a study to determine frequency of burnout among emergency physicians and its effect on their HRQOL.

## METHODS

We conducted a cross-sectional study at Jinnah Hospital Lahore in March 2022. After approval by Institutional Ethical Review Board (288/21/07/22/S1 ERB dated 12/07/22), we included house officers and post graduate trainee doctors working in specialties with emergency services and at least 12 hours a week duty in emergency floor via non-probability purposive sampling. Doctors with less than one-month job duration, those appointed in intensive care unit or outpatient clinic without emergency duties or not willing to participate in study were excluded. Sample size was 142, keeping margin of error 5%, confidence level 80% and expected response distribution of 50% as determined via Raosoft® online calculator.

We used Maslach Burnout Inventory (MBI) for evaluating physicians for burnout. MBI is recognized as the leading measure of burnout and validated by 35+ years of extensive research. The MBI measures burnout as defined by the World Health Organization (WHO) and it is used in 88% of burnout research publications.^13^ It consists of 22 items with Likert scale ranging from never (0) to every day (6). These items represent the three dimensions; EE (Emotional Exhaustion), DP (Depersonalization) and PA (Loss of Personal Accomplishment). For each dimension scores are either low, moderate or high. For EE, score of 0-18 was labelled as low, 19-27 moderate and score of 27 or higher was regarded as high burnout. In DP domain 0-5 was low, 6-9 score was moderate and score of 10 or higher was high burnout. For PA domain, score of 40 or more was low burnout, 34-39 was moderate while score of 33 or less was high burn out. Low scores in all three domains or moderate score in one domain with remaining low scores was regarded as low overall burnout while all domains in moderate, two domains in moderate with low in 3^rd^ or one domain in high with one moderate and one low or low in both was regarded as overall moderate burnout while those with high score in two or more domains were categorized as high burnout.

Instrument used for evaluation of HRQOL was Short Form-36 (SF-36), a questionnaire developed in Medical Outcome Study (MOS) for measuring HRQOL.^14^ It measures quality of life across eight domains, which are both physically and emotionally based and include Physical Functioning (PF), role limitation due to physical health (RP), role limitation due to Emotional Problems (RE), Vitality (VT), General Mental Health (MH), Social Functioning (SF), bodily pain (BP) and General Health perception (GH). Once filled, an aggregate percentage score is produced for each of eight domains measured by SF-36. It is done in two step process. Each of the question responses is related to a pre-coded numeric value.

The response to each question is translated into raw score from 0 to 100, with 0 representing a very low-level quality of life (QOL) and 100 depicting a very positive response to the item. In second step, we took these translated item scores and determined the score for each of the eight domain by adding scores of items related to a domain and dividing it by number of items used. For domain of PF, we added scores of item no 3, 4, 5, 6, 7, 8, 9, 10, 11 and 12 and divided it by 10. Similarly, we added scores of item 13, 14, 15 and 16 and divide it by 4 to determine score of RP, scores of item 17,18,19 gave score of RE, item 23, 27, 29 and 31 gave score for VT, item 24, 25, 26, 28 and 30 calculated score for MH, addition of item 20 and 32 and dividing by two produced score for SF, 21 and 22 items for BP and item 1, 33, 34, 35 and 36 gave score for GH. We also calculated Physical Component Summary (PCS) depicting physical QOL by averaging scores of PF, RP, BP and GH and Mental Component Summary (MCS) representing mental QOL by determining average of SF, RE, MH and VT.

### Statistical Analysis

Data was analyzed using SPSS® 20 (SPSS Inc. Chicago, IL, USA). Variables were expressed as mean ± standard deviation (SD) or in percentage where appropriate for normally distributed variables while median and interquartile range (IQR) for variables not normally distributed. Shapiro-Wilk test was used for checking whether variables were normally distributed or not. We determined Cronbach’s alpha to determine internal consistency of MBI inventory and SF-36 and value above 0.7 was considered satisfactory for study group.

Unpaired two tailed student’s t-test was used to compare mean values among different groups while Mann Whitney U test was used for non-parametric variables. Chi square X^2^ was used for comparing categorical or nominal variables. Analysis of variance (ANOVA) was used to compare HRQOL life scores among study participants with low, moderate and high burnout. P-value of < 0.05 was considered significant for all statistical analysis.

**Table:** ANOVA

		Sum of Squares	df	Mean Square	F	Sig.
Mental component summary	Between Groups	12222.855	2	6111.427	20.033	.000
	Within Groups	44845.610	147	305.072		
	Total	57068.464	149			
Physical component summary	Between Groups	2487.765	2	1243.882	5.743	.004
	Within Groups	31836.778	147	216.577		
	Total	34324.543	149			

## RESULTS

A total of 150 doctors from specialties with emergency service, including house officers (HO) and post graduate residents (PGR) who filled both burnout and SF-36 forms were included in study after informed consent. Mean age was 26.2 (±2.59), male to female ratio 0.78:1 (66/84) and HO to resident ratio was 1.94:1 (99/51). Cronbach’s Alpha was 0.861 and 0.836 for MBI and SF-36 respectively depicting highly consistent data. High burnout among included physicians was identified in 76 (50.7%) participants while 48 (32%) doctors had moderate and 26 (17.3%) had low burnout as assessed by MBI. No difference in severity of burnout in its three domains, (EE), (DP) and (PA) between male and female doctors was noted as shown in Table-I. However, PGRs had less burnout as compared to HO {OR 0.246 (95% CI 0.102-0.593) p-value 0.001} with significant difference in domains of EE {mean MBI score of 27.0(±14.2) in HO as compared to 22.0 (±12.6) in PGRS (p-value 0.03)} and DP {9.51(±6.7%) in HO as compared to 6.4 (±5.9) in PGRs (p-value 0.007)}. MBI items with significant difference between HO and PGRs are shown in Table-II.

**Table-I:** Gender based Distribution of severity of burnout.

Burnout domain	Degree	Total n (% in domain)	Male	Female	P-value
Emotional Exhaustion	Low	53 (35.3)	26	27	0.56
	Moderate	31 (21.7)	14	17	
	High	66 (44)	26	40	
Depersonalization	Low	57 (38)	19	38	0.07
	Moderate	35 (23.3)	20	15	
	High	58 (38.7)	27	31	
Personal achievement	Low	40 (26.7)	18	22	0.98
	Moderate	21 (14)	9	12	
	High	89 (59.3)	39	50	

**Table-II:** Maslach Burnout Inventory items with difference between HO and PGRs.

MBI Item	House officers Mean score (± SD)	PGRs Mean score (± SD)	P-value
	*Range (0-6)*	*Range (0-6)*	
I feel frustrated by my work	3.16(2.1)	2.39(1.8)	0.03
I feel like I am at end of my rope	2.27(2.28)	1.59(1.61)	0.05
I am at end of my patience at the end of my work day	2.55 (2.1)	1.73 (2.04)	0.02
I have impression that my patients make me responsible for their problems	2.44 (1.9)	1.73(1.84)	0.03
Have become insensitive to people since I have been working	2.09 (1.79)	1.33(1.49)	0.01
I am afraid that this job is making me uncaring	1.85(2.06)	1.04(1.41)	0.01
I feel full of energy	3.71(1.92)	3.09 (1.76)	0.05

HRQOL of included doctors was assessed using SF-36. Mean scores in domains were 69.02(±19.63) for PF, 64.5(±34.4) for RP, 55.13(±40.5) in RE, 52.76 (±19.75) in VT, 61.73(±20.1) in MH, 61.61(±23.29) in SF, 66.50(±22.53) in BP and 64.36(±15.17) in GH. Mean score in physical component summary was 64.36(±15.17) and score was 57.18(±19.57) in mental component summary. Male doctors had better scores in VT {57.5(±19) vs 48.98(±19.6) p-value 0.008}, MH {67.09(±18.09) vs 57.5(±20.7) p-value 0.004} and mental component summary {62.06(±18.34) vs 54.47(±19.9) p-value 0.01} than female physicians while PGRs had better scores than HOs in VT {57.2(±19.06) vs 50.4(±19.8) p-value 0.045} and GH {62.05(±22.8) vs 55.95(±12.7) p-value 0.03}.

Low and high burnout was correlated with domains of HRQOL as shown in Table-III. Physicians with high burnout have significantly lower scores in all domains of HRQOL except physical activity domain. Correlation of different grades of burnout with physical and mental component summary is shown in Graph-I. Physicians with high EE score had significantly lower scores in physical component summary {58.9(±15.1) vs 68.5(±14.02) p-value 0.001} and mental component summary {48.18(±17.6) vs 67.2(±18.5) p-value 0.001} as compared to those with low EE score.

**Table-III:** Degree of burnout and domains of Health related quality of life.

	Low burnout	High burnout	P-value
Physical activity	71.63 (21.78)	68.51(19.27)	0.49
Role limitation due to physical health	79.80 (29.17)	61.18(37.28)	0.023
Role limitation due to emotional health	73.07 (38.89)	44.73 (40.57)	0.002
Vitality	69.80 (15.45)	46.51 (20.28)	<0.0001
General mental health	76.15 (14.81)	55 (20.40)	<0.0001
Social functioning	75.67 (20.52)	52.96 (22.34)	<0.0001
Body pains	76.25 (20.02)	62.59 (23.04)	0.008
General health perception	64.23 (10.45)	55.46 (20.96)	0.04
Physical component summary	72.98 (12.01)	61.68 (15.73)	0.001
Mental component summary	73.67 (16.13)	49.80 (18.62)	<0.0001

**Graph-I:**
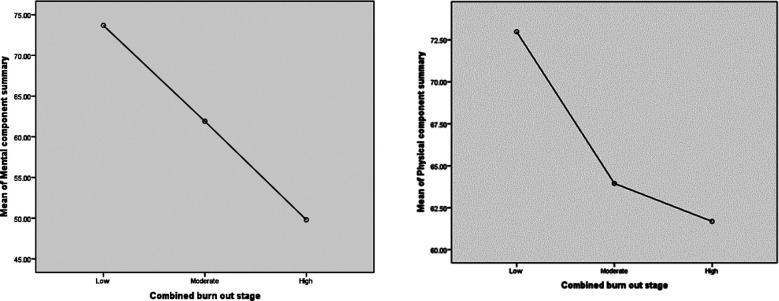
Correlation of severity of burnout and physical and mental component summary.

Participants with high DP scores had lower aggregate in physical component summary {61.28(±15.6) vs 67.51(±15) p-value 0.03} and in mental component summary {50.43(±18.9) vs 63.21(±18.4) p-value <0.0001} as compared to those with low DP score. However, no significant difference in physical component summary scores was noted among doctors with low and high personal achievement burnout scores {66.1(±14.4) vs 62.7(±14.9) p-value 0.23} but mental component summary score was significantly lower in those with high burnout in personal achievement domain {53.4(±18.8) vs 66.5(±19.4) p-value <0.0001) when compared to those with low burnout in personal achievement.

## DISCUSSION

Health is not only the absence of disease but it includes physical, mental and social well-being.^15^ Quality of life is primarily an appraisal of the living environment and gratification of individual in that environment.^16^ We, in this study have explored burnout among young physicians with emergency duties and its impact on their quality of life. We have identified high grade burnout in 76(50.7%) doctors while another 48(32%) had moderate burnout. More than 80% burnout in junior doctors with emergency duties is an alarming figure. In an Egyptian study of physicians and nursing staff working in emergency hospital, 66% had moderate to high grade burnout.^17^ However a canadian study of emergency physicians also noted burnout in more than 90% of study participants similar to more than 80% burnout identified in our study.^11^ Burnout was noted even in 66.7% of general physicians in Egypt and 48.6% of Pakistani nurses.^18^,^19^

We have not found significant gender-based difference in three domains of burnout in our study. Literature too has mixed results as a study of medical residents at General Hospital of Mexico noted higher burnout in females^20^ while a multicenter data of burnout among ICU caregivers has concluded that males have more burnout.^21^ Health related quality of life (HRQOL) scores were low in role limitation due to emotional health 55.13(±40.5), vitality 52.76(±19.75) and mental component summary 57.18(±19.57) among emergency physicians as compared to other domains in our study. Female doctors had significantly lower scores in vitality, general mental health and mental component summary as compared to their male counterpart, a fact also noted in another study of resident physicians by Kassam A et al.^22^

Quality of life scores were significantly lower in all domains of SF-36 proforma in physicians with high burnout score and difference was uniformly noted across all three domains of burnout with markedly poor QOL for highly burned out physicians in vitality, general mental health, social functioning and mental component summary (p-value< 0.0001 in these domains). A study of Greek Health professionals concluded that burnout negatively impacts quality of life in healthcare providers and leads to more sick leaves.^23^ In a study of 16,187 residents, overall burnout was reported to be 51.5% and interestingly emotional exhaustion declined with increasing year of training but depersonalization increased after first year of residency. Scores in Internal Medicine In-training examination were lower for residents with emotional exhaustion and bad quality of life.^24^ However another study identified that emotional exhaustion and depersonalization are associated with younger age and shorter job duration.^25^

Effect of burnout on quality of life can have grave consequences. In a study of 186 physicians of Sedgwick county USA, overall burnout was noted in 49.5% of them but more worrying was the fact that those with burnout had 2.43 times higher prevalence of depression, 1.13 times more suicide ideation and 1.89 times more fatigue as compared to those without burnout.^26^ Burnout is not a sign of brittleness, mental disorder or failure to cope with life, it can be treated and prevented provided we create awareness among health professionals and have systems in place to identify it in health workers and provide needed support and treatment in time to avoid its grave consequences on health related quality of life.^27^

### Limitations of the study

Our study included only physicians of one tertiary care setting which may limit its generalizability. Therefore, the results may not be applicable to physicians working in primary and secondary healthcare setting or those in outpatient clinic due to different working environment and working hours. Apart from compromising HRQOL, burnout can result in poor patient care, reduced work productivity and low job satisfaction which were not evaluated in our study and needs further research to explore these adverse outcomes of burnout.

## CONCLUSION

Physicians working in emergency settings have very high frequency of burnout and it adversely effects their both mental and physical health related quality of life. We therefore recommend institutional policies review to improve working environment and rationalize workload of doctors to reduce burnout along with regular mental health assessment of staff and provision of necessary support and treatment for those suffering from burnout.

### Author’s Contribution:

**SS:** Conception, Design, analysis and interpretation, drafting of article, approval of the version, agreement to be accountable for all aspect.

**AM, YM** and **MF:** Data collection, revising manuscript critically, final approval of the version and agreement to be accountable.
